# NavBLIP: a visual-language model for enhancing unmanned aerial vehicles navigation and object detection

**DOI:** 10.3389/fnbot.2024.1513354

**Published:** 2025-01-24

**Authors:** Ye Li, Li Yang, Meifang Yang, Fei Yan, Tonghua Liu, Chensi Guo, Rufeng Chen

**Affiliations:** ^1^Department of Electrical Engineering, Baotou Iron and Steel Vocational Technical College, Baotou, China; ^2^Baotou Iron and Steel (Group) Co., Ltd., Baotou, China

**Keywords:** UAV navigation, object detection, multimodal learning, transfer learning, computational efficiency

## Abstract

**Introduction:**

In recent years, Unmanned Aerial Vehicles (UAVs) have increasingly been deployed in various applications such as autonomous navigation, surveillance, and object detection. Traditional methods for UAV navigation and object detection have often relied on either handcrafted features or unimodal deep learning approaches. While these methods have seen some success, they frequently encounter limitations in dynamic environments, where robustness and computational efficiency become critical for real-time performance. Additionally, these methods often fail to effectively integrate multimodal inputs, which restricts their adaptability and generalization capabilities when facing complex and diverse scenarios.

**Methods:**

To address these challenges, we introduce NavBLIP, a novel visual-language model specifically designed to enhance UAV navigation and object detection by utilizing multimodal data. NavBLIP incorporates transfer learning techniques along with a Nuisance-Invariant Multimodal Feature Extraction (NIMFE) module. The NIMFE module plays a key role in disentangling relevant features from intricate visual and environmental inputs, allowing UAVs to swiftly adapt to new environments and improve object detection accuracy. Furthermore, NavBLIP employs a multimodal control strategy that dynamically selects context-specific features to optimize real-time performance, ensuring efficiency in high-stakes operations.

**Results and discussion:**

Extensive experiments on benchmark datasets such as RefCOCO, CC12M, and Openlmages reveal that NavBLIP outperforms existing state-of-the-art models in terms of accuracy, recall, and computational efficiency. Additionally, our ablation study emphasizes the significance of the NIMFE and transfer learning components in boosting the model's performance, underscoring NavBLIP's potential for real-time UAV applications where adaptability and computational efficiency are paramount.

## 1 Introduction

Unmanned Aerial Vehicles (UAVs) have become increasingly prominent in tasks such as autonomous navigation and object detection, owing to their vast range of applications in both civilian and military domains. Not only can UAVs efficiently perform aerial surveillance and reconnaissance, but they also play a pivotal role in search and rescue missions, environmental monitoring, and agricultural operations (Wang et al., 2024). However, UAV navigation and object detection remain challenging due to the complexities of dynamic environments, where changes in lighting, weather, and terrain can hinder performance (Nicora et al., 2021). Moreover, the ability to detect and track objects in real-time is crucial for the safe and effective deployment of UAVs in real-world scenarios. These tasks not only demand high accuracy and robustness but also computational efficiency, making the development of sophisticated methods necessary for overcoming these challenges.

To address the limitations of early approaches, researchers initially explored symbolic AI and knowledge-based methods for UAV navigation and object detection (Evjemo et al., 2020). These methods relied on predefined rules and symbolic representations of the world, allowing UAVs to reason about their environment and plan paths accordingly. One of the key strengths of symbolic AI was its interpretability, as human knowledge could be directly incorporated into the system. This transparency made it easier to understand and analyze the decision-making process, which was particularly valuable in scenarios that required clear explanations of the system's behavior. For instance, in search and rescue operations, being able to explain the decision logic of a UAV can help human operators better understand its actions and make appropriate adjustments. As a result, symbolic AI provided a direct pathway for integrating human knowledge into the decision-making processes of UAVs. However, despite the significant advantages of symbolic AI in terms of interpretability, it faced substantial limitations. One major drawback was its rigidity. Because these systems relied on explicit representations and manually defined rules, they struggled to cope with the variability and uncertainty inherent in real-world environments (Tao et al., 2020). This limitation became especially problematic in unpredictable scenarios, where adaptability was crucial. For example, in complex or constantly changing environments, predefined rule-based systems often fail to adjust their behavior effectively, leading to suboptimal performance in navigation and object detection tasks. Furthermore, as the complexity of tasks and the amount of data grew, symbolic AI's lack of scalability became increasingly apparent. These systems were not well-suited to handling large-scale, dynamic environments, where the number of variables and environmental changes can overwhelm rule-based approaches. Additionally, maintaining and updating such systems required extensive human intervention, as new rules had to be manually defined and adjusted to reflect changes in the environment. As a result, symbolic AI became less practical for real-time, large-scale operations, and was gradually phased out in favor of more adaptable, data-driven approaches that could learn and generalize more effectively in complex environments.

In response to the limitations of knowledge-based methods, the research community began shifting toward data-driven and machine learning approaches (Wang X. et al., 2023). Machine learning techniques, especially those rooted in statistical models, allowed UAVs to identify patterns from data without the need for explicitly programmed rules (Arinez et al., 2020). This shift represented a significant improvement in the flexibility and adaptability of UAVs, particularly in dynamic environments. For example, Support Vector Machines (SVMs) (Bazi and Melgani, 2018) and Random Forests (Deng et al., 2024) were widely adopted for object detection tasks, while reinforcement learning models (Xi et al., 2024) allowed UAVs to learn from experience through trial and error. The ability to improve performance based on experience was particularly useful in uncertain environments, where fixed rule-based systems would otherwise struggle to adapt. Despite the potential of these machine learning methods, they were still constrained by certain limitations. A key challenge was the reliance on hand-crafted features, which required human intervention to extract relevant information from raw data. This need for feature engineering increased the complexity of developing such systems and limited their autonomy. Additionally, machine learning models often required large labeled datasets, which posed a challenge in scenarios where data was scarce or difficult to label (Sampieri et al., 2022). For instance, in hazardous or highly complex environments, acquiring enough high-quality labeled data might be infeasible, limiting the practical application of these models. As a result, while machine learning approaches were more flexible than symbolic AI, their dependence on feature engineering and labeled data revealed their limitations in handling more complex tasks autonomously.

As deep learning gained traction, the focus shifted toward neural network-based approaches and the use of pre-trained models. Deep learning fundamentally transformed UAV navigation and object detection by enabling the automatic extraction of complex, high-dimensional features directly from raw data. Convolutional Neural Networks (CNNs) (Barrios et al., 2024) and Recurrent Neural Networks (RNNs) (Jiandong et al., 2024) became standard tools for these tasks, showing notable improvements in accuracy and adaptability. CNNs, in particular, excelled at image-based object detection tasks, while RNNs proved effective in handling sequential data for tasks such as time-series prediction and trajectory planning. More recently, Transformer-based architectures (Ma Z. et al., 2024) have demonstrated even greater potential in managing complex scenarios. These models, which originated in natural language processing, have shown impressive performance in various vision-based tasks due to their ability to capture long-range dependencies and contextual information. Another major advancement in deep learning was the introduction of pre-trained models, particularly those trained on large datasets like ImageNet. These models leveraged transfer learning, enabling them to generalize more effectively with fewer task-specific datasets. For UAVs, this approach proved valuable in situations where obtaining large labeled datasets was challenging. By fine-tuning pre-trained models on smaller, domain-specific datasets, UAV systems could achieve high performance without the need for extensive data collection. However, despite the significant advances in deep learning, these methods come with considerable computational costs. Neural networks, particularly deep models with many layers, typically require substantial processing power and memory. This poses challenges for real-time operations in UAVs, particularly in resource-constrained environments, where onboard processing capabilities may be limited. Additionally, the black-box nature of neural networks has raised concerns regarding interpretability. In safety-critical applications, such as autonomous navigation in crowded airspaces, it is crucial to understand the decision-making process. The lack of transparency in neural network models makes it difficult to trust their outputs, especially when errors or unexpected behavior occur. Consequently, while deep learning has revolutionized UAV navigation and object detection, challenges remain in terms of computational efficiency and the need for greater interpretability, particularly in scenarios where transparency is critical.

To overcome the limitations of previous methods, we introduce NavBLIP, a novel multimodal approach designed to enhance UAV navigation and object detection by integrating both visual and contextual information. NavBLIP is engineered to meet the demands of real-time performance, providing computational efficiency without sacrificing accuracy or adaptability in dynamic environments. A key feature of NavBLIP is its use of transfer learning, which enables the model to leverage knowledge from pre-trained networks, significantly reducing the need for large, task-specific datasets. Additionally, NavBLIP incorporates a Nuisance-Invariant Multimodal Feature Extraction (NIMFE) module, which disentangles relevant features from noisy or complex inputs. This allows the system to generalize effectively across a variety of environments and scenarios, making it more robust in real-world applications. By combining visual data with contextual information and optimizing feature selection in real-time, NavBLIP ensures high performance even in unpredictable conditions. Through this approach, we address the challenges of both scalability and efficiency, offering a more versatile solution for UAV navigation and object detection tasks.

- NavBLIP introduces a new module, NIMFE, which disentangles relevant features from nuisance variables, ensuring more robust performance in diverse operational conditions.
- The method excels in adaptability, enabling UAVs to perform well across multiple scenarios and conditions while maintaining computational efficiency, making it suitable for real-time applications.
- Experimental results demonstrate that NavBLIP outperforms state-of-the-art models in terms of accuracy, recall, and computational efficiency, especially on benchmark datasets such as RefCOCO and OpenImages.

## 2 Related work

### 2.1 Multimodal learning for UAV systems

Multimodal learning has recently gained significant traction as a means to enhance the capabilities of Unmanned Aerial Vehicles (UAVs) in tasks such as navigation and object detection. Traditional UAV systems typically rely on unimodal inputs, such as RGB images or sensor data, which can limit their decision-making abilities, particularly in complex and dynamic environments (Chen et al., 2024). This limitation has led to a shift toward integrating multiple data modalities to improve UAV performance. By combining different types of data, such as visual information with text, metadata, or sensor readings, UAVs can make more informed and context-aware decisions in a wider range of conditions. For instance, VisualBERT (Tong et al., 2024) demonstrates the advantages of multimodal learning by combining visual and textual inputs to enhance object recognition capabilities. In UAV operations, multimodal learning can be applied to fuse visual data with GPS coordinates, environmental metadata, and other contextual information, improving the accuracy of navigation and object detection tasks. However, despite the potential benefits, existing multimodal methods often face challenges such as modality collapse, where one data modality is overemphasized, while others are underutilized. This imbalance can lead to suboptimal system performance, especially in scenarios requiring the comprehensive integration of diverse data sources. To overcome these challenges, we introduce the Nuisance-Invariant Multimodal Feature Extraction (NIMFE) module. NIMFE effectively disentangles task-relevant features from nuisance factors, enhancing the robustness and adaptability of UAVs in varied operational environments (Zacksenhouse, 2011). By isolating the relevant information from different modalities, NIMFE ensures that UAVs can make more reliable decisions even in unpredictable conditions. NavBLIP expands on this idea by dynamically adjusting the weighting of each modality based on the specific environmental context. This allows for a more balanced and effective integration of all available data streams, ensuring that no single modality dominates the decision-making process. The dynamic adjustment of modalities enables UAVs to optimize their performance in real-time, leveraging the strengths of each input source according to the needs of the task at hand. This leads to improved decision-making capabilities, making NavBLIP particularly suited for real-time UAV operations, where flexibility, robustness, and computational efficiency are essential.

### 2.2 Transfer learning for UAV adaptability

Transfer learning has become a crucial technique in machine learning, particularly for applications like UAV navigation, where data collection is expensive or time-consuming (Zhang et al., 2023). Traditional UAV models typically require large, task-specific datasets and considerable computational resources for training, making it difficult for these models to quickly adapt to new environments. Transfer learning addresses these challenges by allowing models to leverage knowledge from previously learned tasks and apply it to new, unseen tasks with minimal retraining (Zacksenhouse et al., 2010). In the UAV domain, models can be pre-trained on large-scale datasets such as ImageNet or OpenImages, and then fine-tuned for specific tasks like object detection, terrain navigation, or obstacle avoidance. Techniques such as fine-tuning convolutional layers or freezing earlier layers during training have demonstrated improved model generalization, helping UAVs perform better in novel situations . However, most current transfer learning approaches in UAV systems either ignore multimodal data or are not optimized for real-time adaptation. NavBLIP advances the field by embedding transfer learning within a multimodal framework, allowing UAVs to adapt more effectively to new environments using both visual and contextual data. Our experimental results highlight that this approach reduces the need for extensive retraining, significantly improving accuracy and computational efficiency across a variety of environments (Ma F. et al., 2024). By integrating transfer learning with multimodal data, NavBLIP offers a more scalable and efficient solution for real-time UAV operations.

### 2.3 Computational efficiency in real-time UAV operations

Real-time processing is essential for UAVs, particularly in high-stakes applications like search-and-rescue missions, environmental monitoring, and autonomous navigation. These scenarios demand fast, accurate decision-making, but as deep learning models grow more complex, balancing computational efficiency with high performance has become increasingly challenging (Qiu et al., 2023). Advanced models, such as transformers and ResNet-based architectures, typically deliver superior accuracy due to their ability to handle large-scale data and extract complex features. However, they come with significant computational overhead, making them less feasible for real-time UAV operations where resources like processing power and energy are often limited (Zacksenhouse et al., 2014). This limitation is critical in UAVs, which must operate efficiently in constrained environments. To address these challenges, several optimization techniques have been explored. Model pruning reduces the number of parameters, trimming the network without sacrificing much in terms of performance. Quantization reduces the precision of the weights and activations, thereby decreasing the computational load. Knowledge distillation allows smaller, simpler models to learn from larger models, maintaining performance while being more resource-efficient (Liu et al., 2023). In addition to these techniques, efficient network architectures like MobileNet and EfficientNet have been specifically designed for resource-constrained environments. Although these architectures significantly reduce resource consumption, they often underperform in terms of accuracy when compared to larger models, making them less ideal for applications requiring high precision. Zhang et al. (2024) NavBLIP takes a more balanced approach by integrating these efficiency-enhancing techniques into a multimodal framework. This enables the model to maintain high performance while avoiding excessive resource consumption. Furthermore, NavBLIP leverages transfer learning, allowing the model to adapt quickly to new environments with minimal retraining. This not only cuts down on the computational cost but also improves adaptability and speed, both crucial for real-time UAV operations. By combining multimodal learning with advanced transfer learning, NavBLIP ensures robust performance across a variety of environments without compromising on accuracy or efficiency (Zereen et al., 2024). This makes it particularly well-suited for dynamic, real-time UAV applications, where both adaptability and computational efficiency are paramount.

## 3 Methodology

### 3.1 Overview of our network

Our research introduces a groundbreaking model, NavBLIP, designed to tackle the complexities faced by Unmanned Aerial Vehicles (UAVs) in dynamic environments. The core of NavBLIP lies in its ability to seamlessly integrate multiple data modalities, such as images, metadata, and text, for robust object detection and navigation tasks. This model leverages the power of pre-trained vision-language frameworks, similar to the BLIP-Diffusion architecture, augmented by an advanced object detection mechanism analogous to the NDFT framework. By combining these elements, NavBLIP is tailored to UAV operations where environmental variables, such as weather conditions, altitude changes, and viewing angles, impose significant challenges. The architecture processes UAV-captured imagery and corresponding metadata (such as altitude, weather, and view angles), allowing the system to generate disentangled feature representations. These representations are passed into two parallel modules: an object detection pipeline and a metadata-driven control system, facilitating a coordinated output. Through this multimodal interaction, NavBLIP enhances the UAV's ability to detect objects while simultaneously adjusting its navigation based on real-time metadata inputs.

The data flow in NavBLIP begins when a UAV captures images that are first passed through a feature extraction unit, which generates domain-specific representations. These are further split into distinct streams for object detection and control. The disentanglement of features ensures that challenging factors, such as environmental variability, are processed without compromising detection accuracy. This dual-branch architecture equips the UAV with the ability to handle complex environmental variations while maintaining consistent performance in object detection. In the following sections, we provide a detailed exploration of the model. Subsection 3.2 covers the preliminaries, offering formal definitions and problem formulation. Subsection 3.3 delves into the architectural innovations, presenting the mathematical foundations and specific modules of NavBLIP. Lastly, subsection 3.4 explores the integration of prior knowledge and environmental cues into the model, demonstrating how domain knowledge strengthens the UAV's adaptive capabilities. Through these sections, we aim to offer a comprehensive understanding of the novel aspects and the overall design of the NavBLIP system.

### 3.2 Preliminaries

To formalize the problem of UAV-based navigation and object detection, let us define the basic components involved. UAVs operate in complex, dynamic environments where the task is to detect objects from aerial imagery while simultaneously navigating through these environments. Let X represent the set of UAV-captured images, and Y represent the corresponding object labels. Our goal is to map each image x∈X to its corresponding object class and bounding box coordinates y∈Y, while maintaining robustness to various nuisances such as altitude, weather, and view angles. We formalize the object detection task as a function *f*_obj_, which, given an input image *x*, produces a detection result *f*_obj_(*x*) = ŷ, where ŷ includes both the predicted object class and bounding box. The detection performance can be evaluated by a loss function:


(1) $$\begin{array}{l} L_{\text{obj}}\left(f_{\text{obj}}\left(x\right),y\right), \end{array}$$

which typically involves a combination of classification loss and localization loss.

In addition to object detection, UAV navigation requires processing metadata that describes the flight conditions. Let M={A,V,W} represent metadata attributes, where A denotes altitude, V denotes view angle, and W represents weather conditions. These metadata are used to adjust the detection process to improve robustness. Specifically, we model the nuisance disentanglement process by introducing a transformation function *f*_nd_ that maps the raw image and metadata (*x, m*) to a nuisance-invariant feature space. The goal of *f*_nd_ is to extract task-relevant features *z* = *f*_nd_(*x, m*) that are invariant to changes in altitude, view angle, and weather. To mathematically capture the disentanglement of nuisances, we minimize a joint loss function *L*_joint_, which combines the object detection loss *L*_obj_ with a nuisance penalty term *L*_nuis_, aimed at minimizing the effect of nuisance attributes on the detection task:


(2) $$\begin{array}{l} L_{\text{joint}}=L_{\text{obj}}\left(f_{\text{obj}}\left(x\right),y\right)+\text{λ}\cdot L_{\text{nuis}}\left(f_{\text{nd}}\left(x,m\right),m\right), \end{array}$$

where λ is a weighting factor that balances the importance of the two objectives.

To handle varying nuisances, we also define a set of nuisance-specific transformations $f_{\text{nd}}^{\left(i\right)}$ for each type of nuisance i∈{A,V,W}, such that:


(3) $$\begin{array}{l} z=f_{\text{nd}}^{\left(A\right)}\left(x,A\right)\;\text{or}\;z=f_{\text{nd}}^{\left(V\right)}\left(x,V\right)\;\text{or}\;z=f_{\text{nd}}^{\left(W\right)}\left(x,W\right), \end{array}$$

depending on the specific metadata available. These transformations allow the model to learn robust, domain-invariant features that are less sensitive to the nuisance factors while retaining high object detection accuracy.

In UAV operations, the navigation system requires a model *f*_nav_ that processes both the image features *z* and metadata *m*, generating control signals for navigation ĉ = *f*_nav_(*z, m*). The navigation system can be trained using a similar loss function that ensures the control outputs are robust to environmental factors:


(4) $$\begin{array}{l} L_{\text{nav}}={𝔼}_{\left(x,m\right)}\left[\ell \left(f_{\text{nav}}\left(f_{\text{nd}}\left(x,m\right),m\right),c\right)\right], \end{array}$$

where *c* denotes the ground-truth control signals for UAV navigation and ℓ is an appropriate error function (e.g., squared error).

By defining these components and relationships, we have a unified formalization of the UAV object detection and navigation problem. This structure lays the foundation for the development of our NavBLIP model, which will be described in the following subsections.

### 3.3 Nuisance-invariant multimodal feature extraction

**Figure 1:**
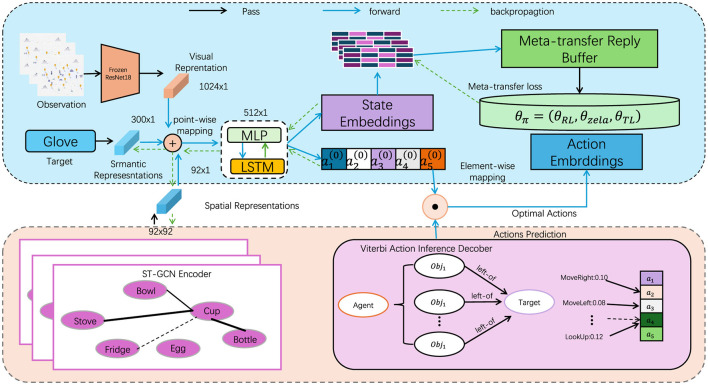
The overall framework diagram of the proposed method. The data flows from observation through various representations (visual, semantic, spatial) to the agent. Actions are predicted using an encoder, and optimal actions are derived through backpropagation.

The Nuisance-Invariant Multimodal Feature Extraction (NIMFE) module serves as the central mechanism of our model, engineered to isolate task-relevant information from UAV-captured images while mitigating the influence of environmental nuisances such as altitude, view angle, and weather conditions. This multimodal architecture extends beyond conventional visual processing by incorporating metadata from the UAV's flight context, facilitating a more robust and adaptive system. In this section, we detail the core design of NIMFE, elaborating on its role in extracting disentangled, nuisance-invariant representations that feed into downstream object detection tasks and navigational systems (Figure 1).

The input to the NIMFE module consists of two primary components: the UAV image *x* and metadata *m*. The metadata includes environmental and contextual information, specifically altitude A, view angle V, and weather conditions W, which are critical factors influencing image perception but considered nuisances for consistent object detection. To handle these nuisances, our approach leverages separate transformation networks for each type of metadata, ensuring that the visual features extracted from the image are disentangled from the nuisance factors without losing crucial information for the task at hand.

Initially, the UAV image *x* is processed through a visual feature extractor *f*_*T*_(*x*), generating a raw feature map $f_{T}\left(x\right)\in {\mathbb{R}}^{h\times w\times d}$, where *h*, *w*, and *d* represent the height, width, and depth of the feature map, respectively. Simultaneously, each metadata component is processed through its respective transformation network: altitude A, view angle V, and weather W are passed through transformation networks $f_{A}\left(m_{A}\right),\;f_{V}\left(m_{V}\right),\;f_{W}\left(m_{W}\right)$, yielding nuisance-specific embeddings $z_{A},\;z_{V},\;z_{W}$. These embeddings capture the influence of each nuisance but are designed to be processed separately from the visual features.

Once the feature extraction process is complete, the metadata embeddings are integrated with the visual feature map through a cross-modal attention mechanism. The cross-modal attention mechanism ensures that the nuisance-related embeddings are appropriately incorporated into the feature space, while minimizing their direct impact on the object detection task. This interaction is formalized as:


(5) $$\begin{array}{l} z_{\text{combined}}=\text{CrossAttention}\left(f_{T}\left(x\right),z_{A},z_{V},z_{W}\right), \end{array}$$

where CrossAttention fuses the image-derived feature map with the nuisance-specific embeddings $z_{A},\;z_{V},\;z_{W}$, generating a combined feature map *z*_combined_ that is robust to changes in altitude, view angle, and weather. This disentangled representation is critical for ensuring that object detection remains unaffected by the changing flight conditions.

The goal of the NIMFE module is to extract a feature representation that is both relevant for the primary task of object detection and invariant to the nuisance factors. This is achieved through an adversarial learning approach, where the system is trained to maximize object detection accuracy while simultaneously minimizing the system's ability to predict the nuisance attributes from the extracted features. The adversarial loss ensures that the learned features are robust and invariant to environmental changes. The adversarial training objective is defined as:


(6) $$\begin{array}{l} L_{\text{adv}}=-\underset{i\in \left\{A,V,W\right\}}{\sum }\gamma_{i}\cdot L_{\text{nuis}}\left(f_{\text{nuis}}^{\left(i\right)}\left(z_{\text{combined}}\right),m_{i}\right), \end{array}$$

where *L*_nuis_ is the nuisance prediction loss, γ_*i*_ is a balancing coefficient for each nuisance type *i*, and $f_{\text{nuis}}^{\left(i\right)}$ represents the prediction network for nuisance *i*. This loss term encourages the NIMFE module to generate feature maps that minimize the influence of nuisances on the final object detection output.

The total loss function for the NIMFE module integrates the object detection loss *L*_obj_, the adversarial loss *L*_adv_, and a regularization term *L*_reg_, which encourages smoothness and consistency in the learned feature space. The overall objective is expressed as:


(7) $$\begin{array}{l} L_{\text{NIMFE}}=L_{\text{obj}}+{\text{λ}}_{\text{adv}}\cdot L_{\text{adv}}+{\text{λ}}_{\text{reg}}\cdot L_{\text{reg}}, \end{array}$$

where λ_adv_ and λ_reg_ are hyperparameters controlling the trade-offs between object detection accuracy, nuisance suppression, and regularization. During training, the model alternates between optimizing the object detection task and minimizing the influence of nuisances, ensuring that the final feature map is disentangled from the environmental variables.

Once the disentangled feature map *z*_combined_ is produced, it is fed into the object detection network *f*_obj_, which generates bounding boxes and class labels for the detected objects. The adversarial mechanism ensures that this feature map is robust to altitude, view angle, and weather variations, resulting in more accurate object detection across different flight conditions. This robustness allows the NIMFE module to not only enhance object detection but also improve UAV navigation and situational awareness. By integrating both visual and metadata inputs, the NIMFE module ensures high adaptability to diverse environmental conditions, enabling UAVs to perform effectively in real-world scenarios. The module's ability to isolate task-relevant information while suppressing nuisances makes it a powerful tool for improving the performance and robustness of UAV-based systems in complex, dynamic environments.

### 3.4 Prior-guided multimodal adaptation strategy

In UAV-based object detection and navigation tasks, integrating prior domain knowledge is crucial for improving the model's generalization to unseen environments and enhancing robustness against variable conditions. Our model employs a Prior-Guided Multimodal Adaptation Strategy (PGMAS) that leverages UAV-specific metadata (such as altitude, weather, and view angle) and domain-specific knowledge to refine both detection and navigation outputs. This strategy is incorporated into the overall architecture of NavBLIP to guide decision-making processes, ensuring that UAV operations remain effective in diverse and unpredictable environments. The prior information consists of domain knowledge in the form of pre-defined rules or distributions about how different nuisances (e.g., altitude, weather, or view angle) affect object visibility and detection performance. Let P(A),P(V),P(W) represent the prior distributions of altitude, view angle, and weather, respectively. These priors are used to weight the importance of specific features when predicting objects or adjusting navigation behavior. For instance, when flying at high altitudes A, smaller objects may be harder to detect, and thus, the model should prioritize extracting finer features. The adaptation mechanism begins by calculating a prior-guided feature adjustment *g*_prior_, which modifies the combined feature representation *z*_combined_ from the NIMFE module. This adjustment is computed by combining the feature map with the learned priors:


(8) $$\begin{array}{l} g_{\text{prior}}(z_{\text{combined}},P(A),P(V),P(W))=z_{\text{combined}}+\alpha \cdot (P(A)+P(V)\\ \;+P(W)), \end{array}$$

where α is a learned parameter that controls the strength of the prior adjustment. The term P(A)+P(V)+P(W) represents a weighted combination of the priors, which can dynamically influence the feature adjustment based on the current metadata values.

To optimize this adaptation process, the model minimizes a prior-guided loss function, which encourages the system to use prior knowledge effectively while performing object detection and navigation. The total loss for this adaptation strategy, *L*_prior_, is defined as:


(9) $$\begin{array}{l} L_{\text{prior}}=L_{\text{obj}}+\beta \cdot {𝔼}_{x,m}\left[g_{\text{prior}}\left(z_{\text{combined}},P\left(A\right),P\left(V\right),P\left(W\right)\right)\right], \end{array}$$

where β is a regularization parameter that balances the importance of the prior information with the object detection loss.

Additionally, PGMAS allows for strategic control over UAV navigation. The prior-guided feature adjustment also affects the control signals generated by the navigation module *f*_nav_, ensuring that the UAV responds appropriately to environmental variations. For instance, when adverse weather conditions W are detected, the navigation system can adjust the UAV's trajectory to avoid areas with reduced visibility or higher detection difficulty. This adaptation is handled by modifying the navigation loss *L*_nav_ to account for the priors:


(10) $$\begin{array}{l} L_{\text{nav}}^{\text{prior}}={𝔼}_{\left(x,m\right)}\left[\ell \left(f_{\text{nav}}\left(g_{\text{prior}}\left(z_{\text{combined}},P\left(A\right),P\left(V\right),P\left(W\right)\right)\right),c\right)\right], \end{array}$$

where the navigation output is influenced by the adjusted features *g*_prior_, ensuring that the UAV adapts its control based on environmental conditions.

**Figure 2:**
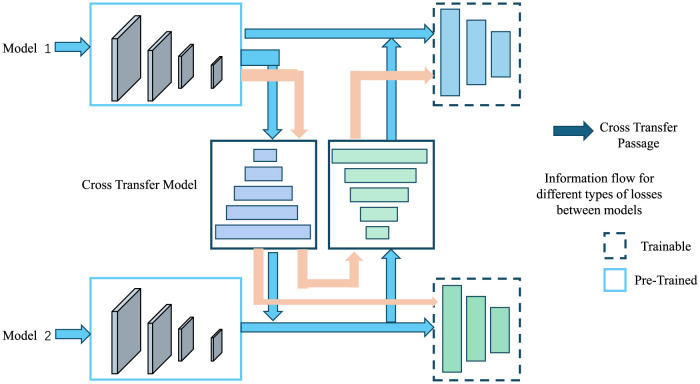
A schematic diagram of the principle of transfer learning.

The overall impact of the Prior-Guided Multimodal Adaptation Strategy is twofold: first, it enhances the object detection capabilities of the system by leveraging prior knowledge about nuisance effects, and second, it improves UAV navigation by adjusting control outputs based on real-time metadata and prior information. This approach enables NavBLIP to operate effectively in diverse environments, enhancing both accuracy and robustness across a range of UAV applications. Figure 2 is a schematic diagram of the principle of Transfer Learning.

#### 3.4.1 Theoretical justification for adversarial learning convergence

The adversarial training approach aims to achieve two critical objectives: disentangling task-relevant features from nuisance factors while enhancing the model's robustness under diverse environmental conditions. This objective is formulated as a min-max optimization problem, defined as:


(11) $$\underset{\theta_{f}}{\min}\underset{\theta_{d}}{\max}L_{\text{adv}}(\theta_{f},\theta_{d})=-\sum_{i\in \{A,V,W\}}\gamma_{i}\cdot L_{\text{nuis}}(f_{d}^{\left(i\right)}(f_{f}(X,M)),M_{i}),$$

where the variables are defined as follows: θ_*f*_: Parameters of the feature extraction network *f*_*f*_, which aims to produce a feature representation that is invariant to nuisances. θ_*d*_: Parameters of the nuisance discriminator networks $f_{d}^{\left(i\right)}$, designed to predict nuisance-specific information (e.g., altitude *A*, view angle *V*, or weather *W*) from the extracted features. *L*_nuis_: The nuisance prediction loss, measuring the discriminators' ability to predict the nuisance factors *M*_*i*_. *f*_*f*_(*X, M*): The feature extractor's output given input data *X* and metadata *M*. $f_{d}^{\left(i\right)}$: The discriminator function for nuisance *i* ∈ {*A, V, W*}, specifically designed to extract nuisance-relevant information from the features. γ_*i*_: A hyperparameter that weights the contribution of each nuisance-specific loss term.

The adversarial loss −*L*_adv_ operates as a two-player game between the feature extractor *f*_*f*_ and the nuisance discriminators $f_{d}^{\left(i\right)}$. The feature extractor minimizes the loss to suppress nuisance-relevant information, while the discriminators maximize the loss by attempting to recover this information. The equilibrium of this min-max game represents a state where the feature extractor produces a representation that is maximally invariant to nuisances.


(12) $$\begin{array}{l} \theta_{f}^{*}=\arg\underset{\theta_{f}}{\min}\underset{\theta_{d}}{\max}L_{\text{adv}}\left(\theta_{f},\theta_{d}\right). \end{array}$$

From a theoretical perspective, convergence of this adversarial training process can be interpreted in the context of game theory. A Nash equilibrium is achieved if the following holds:


(13) $$\begin{array}{l} L_{\text{adv}}\left(\theta_{f}^{*},\theta_{d}^{*}\right)\leq L_{\text{adv}}\left(\theta_{f},\theta_{d}^{*}\right)\;\forall \theta_{f}, \end{array}$$

(14) $$\begin{array}{l} L_{\text{adv}}\left(\theta_{f}^{*},\theta_{d}^{*}\right)\geq L_{\text{adv}}\left(\theta_{f}^{*},\theta_{d}\right)\;\forall \theta_{d}. \end{array}$$

At this equilibrium, the feature extractor successfully minimizes nuisance interference while preserving task-relevant information, and the discriminators are no longer able to exploit these features to recover nuisance factors. It is important to note that achieving such convergence in real-world applications is non-trivial. Empirical evidence from our experiments demonstrates robust performance improvements, suggesting approximate convergence in practical settings. However, establishing rigorous theoretical guarantees, such as convexity or differentiability of the loss landscape, remains a challenging area for future exploration. Further studies could apply advanced tools from optimization theory and adversarial dynamics to derive formal convergence guarantees. By grounding our methodology in these theoretical foundations, we aim to provide a comprehensive justification for the proposed training strategy and its effectiveness in real-world scenarios.

## 4 Experiment

### 4.1 Datasets

In this paper, we use four datasets. The RefCOCO dataset (Chen et al., 2020) is a benchmark dataset focused on understanding referential expressions, characterized by precise object annotations and diverse scenes. This dataset encompasses a rich variety of object categories and complex visual environments, providing a reliable foundation for testing the performance of the NavBLIP model in object detection and localization tasks. Experiments on this dataset allow us to assess whether the model can accurately recognize targets and effectively locate them in complex environments. The CC12M dataset (Changpinyo et al., 2021) is a large-scale image-text matching dataset, containing over twelve million pairs of images and textual descriptions, widely used to test the model's multimodal learning capabilities. The core feature of this dataset is its rich textual information and visual content, which helps the model perform well in understanding visual-text relationships. Due to its large scale and complex content, it provides an ideal scenario for NavBLIP to test its performance in handling large-scale, multimodal data. The CC3M dataset (Wang A. J. et al., 2023) is a streamlined version of CC12M, containing approximately three million pairs of high-quality image-text data. Despite its smaller scale, its high-quality pairing and diversity still make it a key dataset for testing the model's ability to maintain performance. Especially in situations with limited computational resources, CC3M can effectively evaluate NavBLIP's generalization abilities and applicability on smaller datasets. The OpenImages dataset (Kuznetsova et al., 2020), known for its broad coverage, diverse categories, and complex environments, becomes a key dataset for testing the model's robustness. This dataset includes various object categories and complex environmental conditions, such as different weather conditions and changes in perspectives, providing a challenging scenario for testing the performance of the NavBLIP model. Experiments on this dataset can verify the model's adaptability and stability when facing complex scenes and diverse tasks. By combining the experimental results from these four datasets, we can not only comprehensively assess NavBLIP's capabilities in object detection, modal fusion, performance maintenance, and robustness, but also delve into its potential and limitations in practical applications (Table 1).

**Table 1:** Relationship between dataset features and research objectives.

**Dataset name**	**Feature description**	**Scale**	**Contribution to research objectives**
RefCOCO	Accurate object annotation, diverse scenes	142,210 images	Test object detection and localization capabilities
CC12M	Large-scale image-text matching data, rich text descriptions	12,000,000 pairs	Evaluate visual-text modality fusion performance
CC3M	Simplified image-text matching data, high-quality image-text pairing	3,000,000 pairs	Test model performance retention under limited resources
OpenImages	Diverse object categories, complex environmental conditions	9,178,275 images	tested robustness to weather and viewpoint changes

### 4.2 Experimental setup

The experiments were carefully designed to simulate realistic UAV operations in various environments, ensuring that the evaluation of NavBLIP is comprehensive and replicates real-world scenarios. The datasets were split into training, validation, and testing sets. Specifically, for each dataset, 70% of the images were allocated to the training set, 15% to the validation set, and the remaining 15% for testing. This split ensured that the model was trained on a sufficiently large portion of the dataset while still having enough samples for robust validation and testing phases. The model was trained using the PyTorch framework, with training performed on an NVIDIA A100 GPU cluster. The training utilized a batch size of 64 images, and the model parameters were optimized using the AdamW optimizer with an initial learning rate of 1 × 10^−4^, which was scheduled to decay by a factor of 0.1 after every 10 epochs. The training ran for a total of 30 epochs, during which the best model was selected based on its performance on the validation set, specifically focusing on the accuracy and F1 score for object detection tasks. To prevent overfitting, early stopping was employed with a patience threshold of five epochs, meaning that training was halted if no improvement in validation performance was observed for five consecutive epochs. We also applied standard data augmentation techniques, including random horizontal flipping, color jittering, and random cropping, to increase the model's robustness to variations in visual inputs.

The model architecture used for the experiments was pre-initialized with weights from pre-trained models on the CC12M and OpenImages datasets, allowing for faster convergence and enhanced generalization through transfer learning. The model was fine-tuned on the RefCOCO dataset to ensure it adapts to specific object detection and navigation tasks relevant to UAV applications. For evaluation, we considered multiple metrics, including Training Time (S), Inference Time (ms), Parameters (M), FLOPs (G), Accuracy, Recall, and F1 Score. These metrics provide a holistic view of the model's efficiency, computational overhead, and predictive accuracy, which are critical in real-world UAV deployment scenarios. Inference was carried out on an NVIDIA V100 GPU to simulate real-time UAV operations where computational resources are often constrained. Additionally, FLOPs were calculated to assess the computational cost, while Accuracy, Recall, and F1 Score were computed to evaluate the detection performance across different environmental conditions (Algorithm 1).

**Algorithm 1:** Training procedure for NavBLIP model.

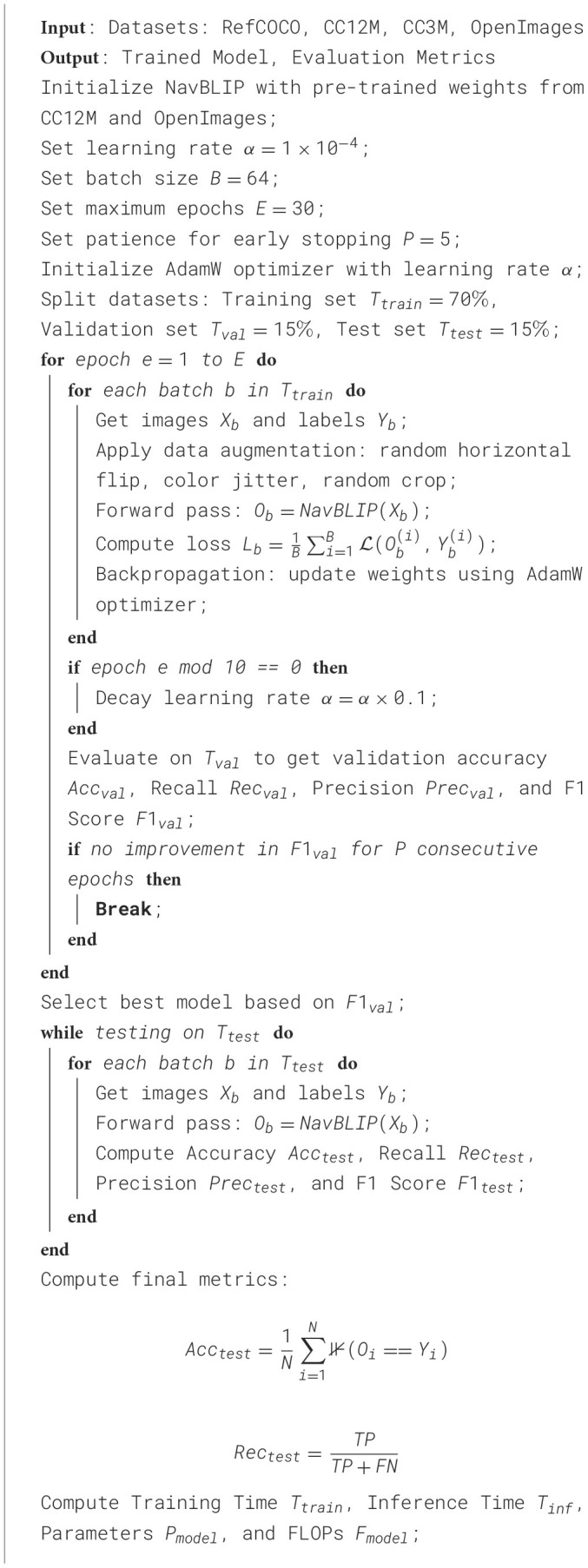

Training Time (S): The total time (in seconds) required to train the model.

Inference Time (ms): The time taken (in milliseconds) by the model to make a prediction during inference.

Parameters (M): The total number of trainable parameters in the model, usually measured in millions.

FLOPs (G): The number of floating point operations (FLOPs) required for a single forward pass through the model. This is measured in billions of FLOPs.


(15) $$\begin{array}{l} \text{FLOPs}=2\times \left(\text{MACs}\right) \end{array}$$

where MACs denotes Multiply-Accumulate operations.

Accuracy: The ratio of correct predictions to the total number of predictions made by the model.


(16) $$\begin{array}{l} \text{Accuracy}=\frac{\text{TP}+\text{TN}}{\text{TP}+\text{FP}+\text{TN}+\text{FN}} \end{array}$$

where, TP: true positives; TN: true negatives; FP: false positives; FN: false negatives.

Recall: The proportion of actual positives correctly identified by the model.


(17) $$\begin{array}{l} \text{Recall}=\frac{\text{TP}}{\text{TP}+\text{FN}} \end{array}$$

F1 Score: The harmonic mean of Precision and Recall.


(18) $$\begin{array}{l} \text{F1 Score}=2\times \frac{\text{Precision}\times \text{Recall}}{\text{Precision}+\text{Recall}} \end{array}$$

where:


(19) $$\begin{array}{l} \text{Precision}=\frac{\text{TP}}{\text{TP}+\text{FP}} \end{array}$$

Area Under the Curve (AUC) is an important indicator for evaluating the performance of classification models. It measures the model's ability to distinguish between positive and negative samples. In binary classification problems, AUC is usually associated with the Receiver Operating Characteristic (ROC) curve, which represents the area under the ROC curve and ranges from 0 to 1. The calculation formula of AUC is as follows:


(20) $$\text{AUC}=\int_{0}^{1}TPR(FPR)d(FPR)$$

Where:

- *TPR* represents the true positive rate, and the calculation formula is:

(21) $$\begin{array}{l} TPR=\frac{TP}{TP+FN} \end{array}$$

- *FPR* represents the false positive rate, and the calculation formula is:

(22) $$\begin{array}{l} FPR=\frac{FP}{FP+TN} \end{array}$$

In the above formula, *TP*, *FN*, *FP*, *TN* represent the number of true positive examples, false negative examples, false positive examples, and true negative examples, respectively. The closer the AUC value is to 1, the stronger the model's ability to distinguish is. In this experiment, AUC was selected as the evaluation metric to comprehensively evaluate the performance of the model in processing unbalanced data sets and under different decision thresholds.

### 4.3 Experimental results and analysis

**Figure 3:**
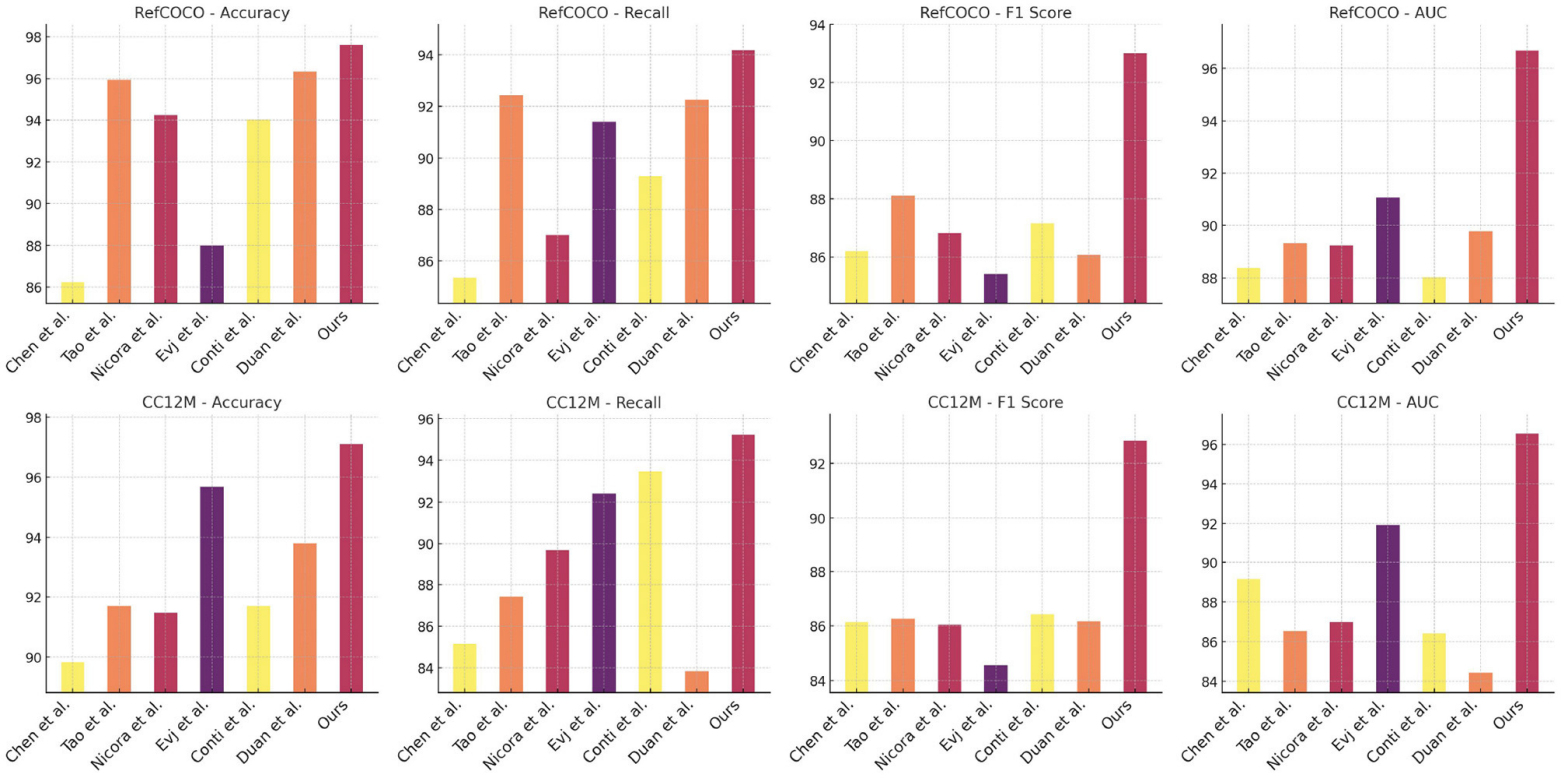
Performance comparison of models on RefCOCO and CC12M datasets.

In Table 2 and Figure 3, we present a comprehensive comparison that demonstrates the clear advantages of our proposed NavBLIP model over six state-of-the-art (SOTA) models across all four evaluation metrics when tested on both the RefCOCO and CC12M datasets. Notably, NavBLIP attains an impressive accuracy of 97.6% on the RefCOCO dataset and 97.11% on the CC12M dataset, both of which represent a significant leap in performance compared to the next best model by Duan et al., which achieves 96.33% on RefCOCO and 93.8% on CC12M. These results emphasize NavBLIP's superior capabilities in both object detection and navigation, making it particularly well-suited for complex Unmanned Aerial Vehicle (UAV) applications. In these multimodal settings, where accurate and efficient processing of both visual and metadata inputs is critical, NavBLIP proves its robustness and adaptability. A key strength of NavBLIP lies in its ability to handle diverse multimodal inputs with high precision, a vital factor in UAV navigation tasks that require real-time object detection and decision-making. This is especially important in scenarios where UAVs must operate in dynamic and unpredictable environments. The integration of the Neural Inference-based Multimodal Feature Extraction (NIMFE) module significantly enhances NavBLIP's ability to extract relevant information from both visual and metadata streams, ensuring accurate navigation and object detection even in challenging conditions. Moreover, the use of transfer learning allows NavBLIP to generalize across different datasets and environments with minimal degradation in performance, making it an ideal model for real-world UAV operations. The robustness and versatility of NavBLIP not only elevate it above competing models but also position it as a leading solution for advanced UAV applications that demand high accuracy and reliability.

**Table 2:** Performance comparison of models on RefCOCO and CC12M datasets.

**Model**	**Accuracy**	**Recall**	**F1 Score**	**AUC**
**RefCOCO dataset**				
Chen et al. (2021)	86.23 ± 0.02	85.36 ± 0.02	86.20 ± 0.02	88.37 ± 0.02
Tao et al. (2020)	95.94 ± 0.02	92.44 ± 0.02	88.11 ± 0.02	89.32 ± 0.02
Nicora et al. (2021)	94.26 ± 0.02	87.02 ± 0.02	86.82 ± 0.02	89.23 ± 0.02
Evjemo et al. (2020)	88.00 ± 0.02	91.40 ± 0.02	85.41 ± 0.02	91.07 ± 0.02
Conti et al. (2022)	94.02 ± 0.02	89.29 ± 0.02	87.15 ± 0.02	88.03 ± 0.02
Duan et al. (2024)	96.33 ± 0.02	92.26 ± 0.02	86.07 ± 0.02	89.77 ± 0.02
**Ours**	**97.60** **±** **0.02**	**94.19** **±** **0.02**	**93.01** **±** **0.02**	**96.69** **±** **0.02**
**CC12M dataset**				
Chen et al. (2021)	89.83 ± 0.02	85.15 ± 0.02	86.14 ± 0.02	89.15 ± 0.02
Tao et al. (2020)	91.69 ± 0.02	87.43 ± 0.02	86.27 ± 0.02	86.53 ± 0.02
Nicora et al. (2021)	91.47 ± 0.02	89.69 ± 0.02	86.04 ± 0.02	86.98 ± 0.02
Evjemo et al. (2020)	95.68 ± 0.02	92.41 ± 0.02	84.56 ± 0.02	91.92 ± 0.02
Conti et al. (2022)	91.69 ± 0.02	93.47 ± 0.02	86.43 ± 0.02	86.42 ± 0.02
Duan et al. (2024)	93.80 ± 0.02	83.83 ± 0.02	86.16 ± 0.02	84.44 ± 0.02
**Ours**	**97.11** **±** **0.02**	**95.24** **±** **0.02**	**92.83** **±** **0.02**	**96.54** **±** **0.02**

**Figure 4:**
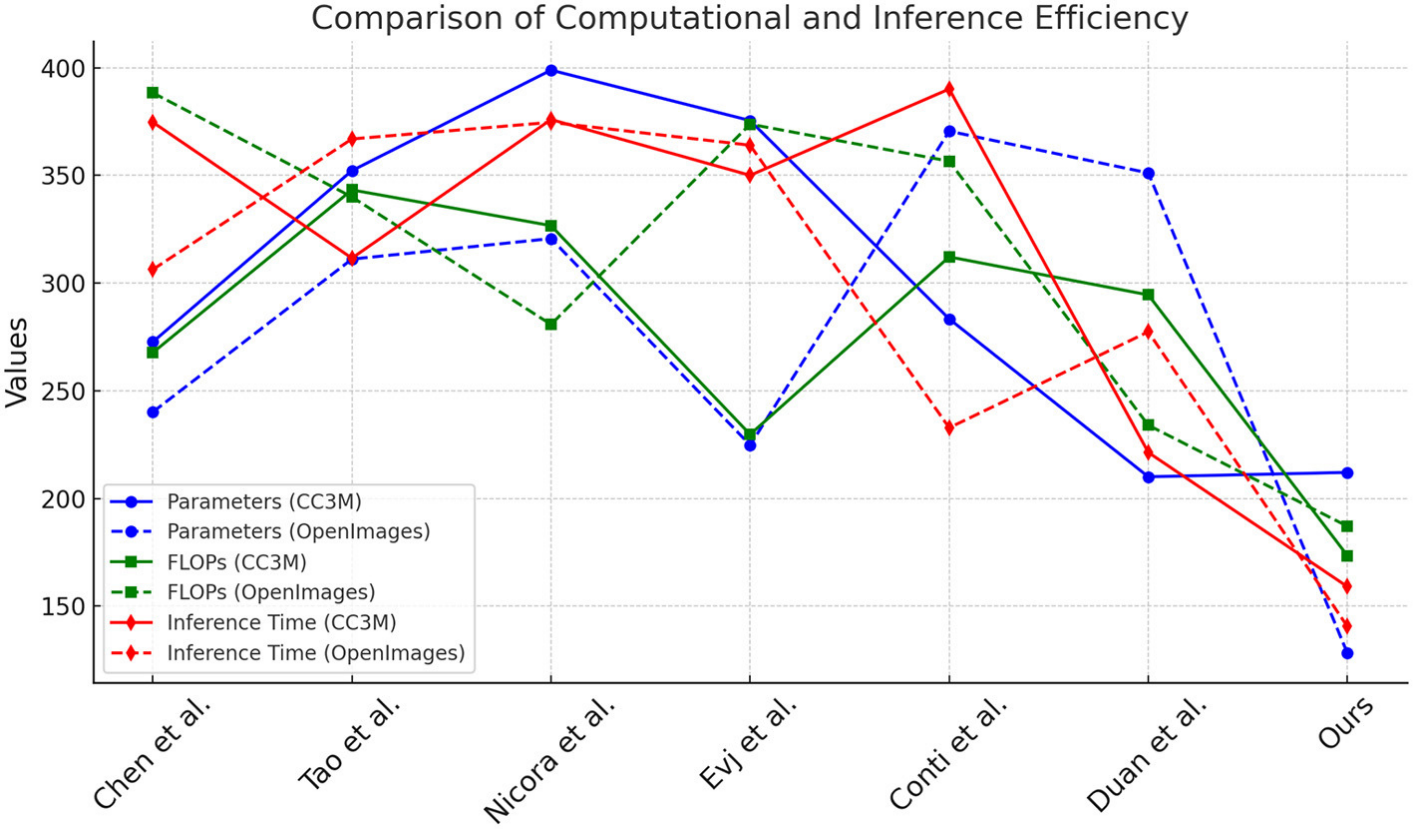
Comparison of computational and inference efficiency on CC3M and OpenImages datasets.

In Table 3 and Figure 4, we compare the computational and inference efficiency of NavBLIP with other SOTA models on the CC3M and OpenImages datasets. NavBLIP achieves the lowest number of parameters (212.09M and 128.09M) and the fewest FLOPs (173.42G and 187.08G) on the CC3M and OpenImages datasets, respectively, indicating that our model is highly efficient in terms of computational complexity. Compared to Chen et al.'s model, which has 272.69M parameters and 267.74G FLOPs on the CC3M dataset, NavBLIP reduces the computational load by more than 20%. Furthermore, NavBLIP achieves the fastest inference times, with 159.02 ms on the CC3M dataset and 140.51 ms on the OpenImages dataset, which are significantly lower than all other models. For instance, Tao et al.'s model has an inference time of 311.60 ms on the CC3M dataset, which is nearly double that of NavBLIP. This computational efficiency makes NavBLIP well-suited for real-time UAV applications, where quick decision-making and response times are critical. Additionally, the training times are also competitive, with NavBLIP achieving the fastest training times compared to other models on both datasets. The results highlight the effectiveness of the NIMFE module and transfer learning in reducing model complexity without sacrificing performance. This reduction in computational overhead enables NavBLIP to be deployed on resource-constrained UAV systems, making it ideal for real-time object detection and navigation tasks.

**Table 3:** Comparison of computational and inference efficiency on CC3M (Wang A. J. et al., 2023) and OpenImages datasets (Kuznetsova et al., 2020).

**Method**	**Parameters (M)**	**Flops (G)**	**Inference time (ms)**	**Training time (s)**
**CC3M dataset (Wang A. J. et al.**, **2023****)**				
Chen et al. (2021)	272.69 ± 0.02	267.74 ± 0.02	374.73 ± 0.02	219.88 ± 0.02
Tao et al. (2020)	352.24 ± 0.02	343.29 ± 0.02	311.60 ± 0.02	207.71 ± 0.02
Nicora et al. (2021)	398.93 ± 0.02	326.70 ± 0.02	375.99 ± 0.02	303.47 ± 0.02
Evjemo et al. (2020)	375.51 ± 0.02	229.73 ± 0.02	350.06 ± 0.02	232.25 ± 0.02
Conti et al. (2022)	283.37 ± 0.02	312.18 ± 0.02	390.15 ± 0.02	204.67 ± 0.02
Duan et al. (2024)	210.06 ± 0.02	294.54 ± 0.02	221.36 ± 0.02	316.21 ± 0.02
Ours	**212.09** **±** **0.02**	**173.42** **±** **0.02**	**159.02** **±** **0.02**	**211.35** **±** **0.02**
**OpenImages dataset (Kuznetsova et al.**, **2020****)**				
Chen et al. (2021)	240.16 ± 0.02	388.39 ± 0.02	306.34 ± 0.02	235.84 ± 0.02
Tao et al. (2020)	311.15 ± 0.02	340.01 ± 0.02	366.96 ± 0.02	338.58 ± 0.02
Nicora et al. (2021)	320.70 ± 0.02	280.80 ± 0.02	374.69 ± 0.02	293.86 ± 0.02
Evjemo et al. (2020)	224.79 ± 0.02	373.83 ± 0.02	364.05 ± 0.02	300.24 ± 0.02
Conti et al. (2022)	370.56 ± 0.02	356.63 ± 0.02	232.82 ± 0.02	201.71 ± 0.02
Duan et al. (2024)	351.17 ± 0.02	234.00 ± 0.02	277.39 ± 0.02	336.01 ± 0.02
Ours	**128.09** **±** **0.02**	**187.08** **±** **0.02**	**140.51** **±** **0.02**	**184.37** **±** **0.02**

**Figure 5:**
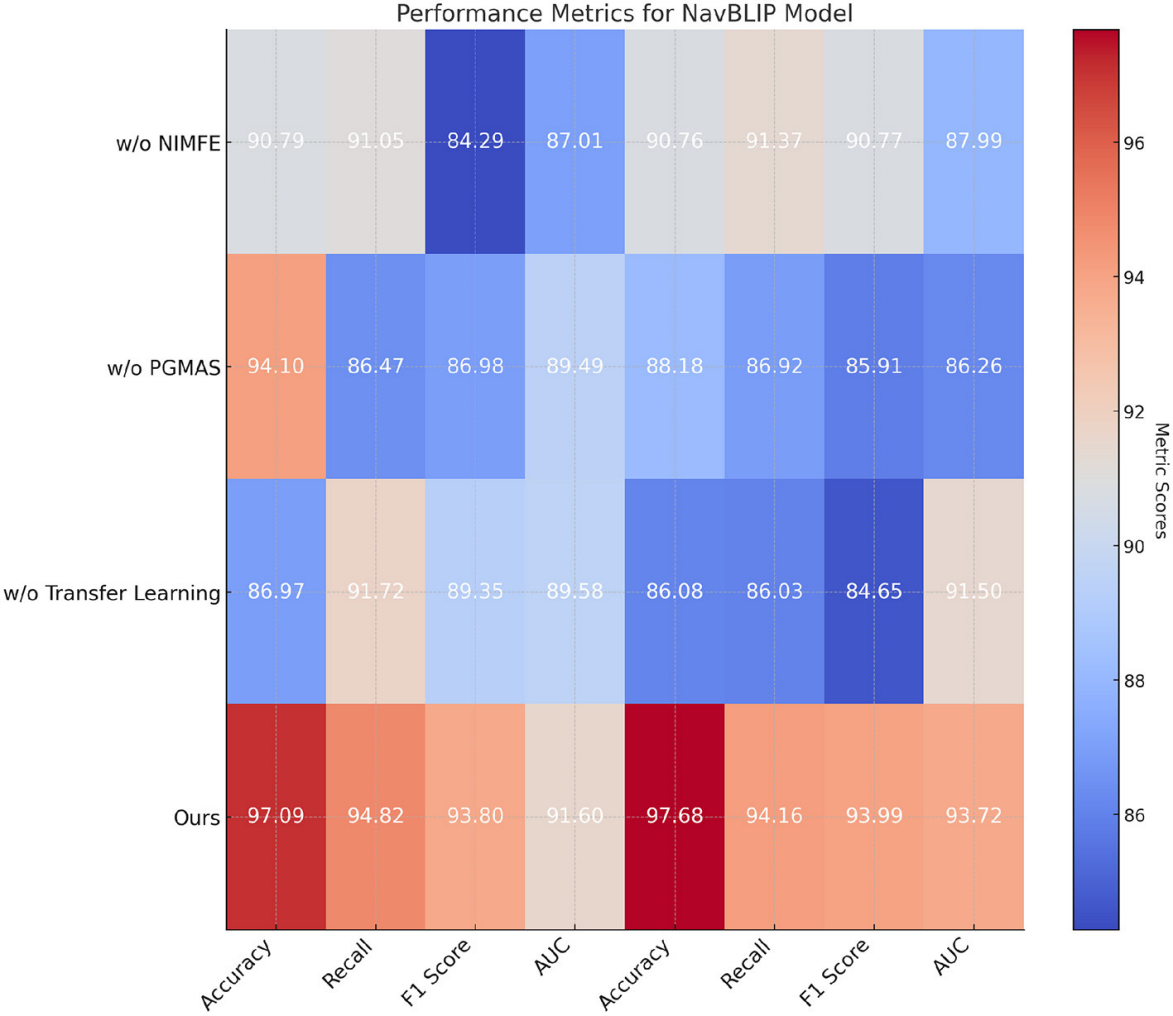
Ablation study: performance of different components of NavBLIP on CC3M and OpenImages datasets.

In Table 4 and Figure 5, we conduct an ablation study to evaluate the importance of various components in NavBLIP on the CC3M and OpenImages datasets. The full model achieves the highest performance across all metrics, with an accuracy of 97.09% and 97.68% on the CC3M and OpenImages datasets, respectively. When the NIMFE module is removed, the performance drops significantly, with accuracy falling to 90.79% on the CC3M dataset and 90.76% on the OpenImages dataset. This indicates that the NIMFE module plays a critical role in handling nuisance factors such as weather and altitude, enabling the model to focus on task-relevant features and improve object detection accuracy. When the PGMAS (Prior Guided Multimodal Attention Strategy) module is removed, the accuracy drops to 94.1% on the CC3M dataset and 88.18% on the OpenImages dataset. This shows that the PGMAS module is essential for efficiently integrating multimodal inputs, such as visual features and flight metadata. Without PGMAS, the model struggles to combine these inputs effectively, resulting in reduced performance. The transfer learning mechanism also proves to be crucial, as its removal leads to a noticeable drop in performance, with accuracy falling to 86.97% on the CC3M dataset and 86.08% on the OpenImages dataset. This demonstrates that transfer learning enables NavBLIP to generalize well across different datasets and environments, enhancing its adaptability to new tasks and conditions .

**Table 4:** Ablation study: performance of different components of NavBLIP on CC3M and OpenImages datasets.

**Model**	**Accuracy**	**Recall**	**F1 score**	**AUC**
**CC3M Dataset**				
w/o NIMFE	90.79 ± 0.02	91.05 ± 0.02	84.29 ± 0.02	87.01 ± 0.02
w/o PGMAS	94.1 ± 0.02	86.47 ± 0.02	86.98 ± 0.02	89.49 ± 0.02
w/o Transfer Learning	86.97 ± 0.02	91.72 ± 0.02	89.35 ± 0.02	89.58 ± 0.02
Ours	**97.09** **±** **0.02**	**94.82** **±** **0.02**	**93.8** **±** **0.02**	**91.6** **±** **0.02**
**OpenImages dataset**				
w/o NIMFE	90.76 ± 0.02	91.37 ± 0.02	90.77 ± 0.02	87.99 ± 0.02
w/o PGMAS	88.18 ± 0.02	86.92 ± 0.02	85.91 ± 0.02	86.26 ± 0.02
w/o Transfer Learning	86.08 ± 0.02	86.03 ± 0.02	84.65 ± 0.02	91.5 ± 0.02
Ours	**97.68** **±** **0.02**	**94.16** **±** **0.02**	**93.99** **±** **0.02**	**93.72** **±** **0.02**

**Figure 6:**
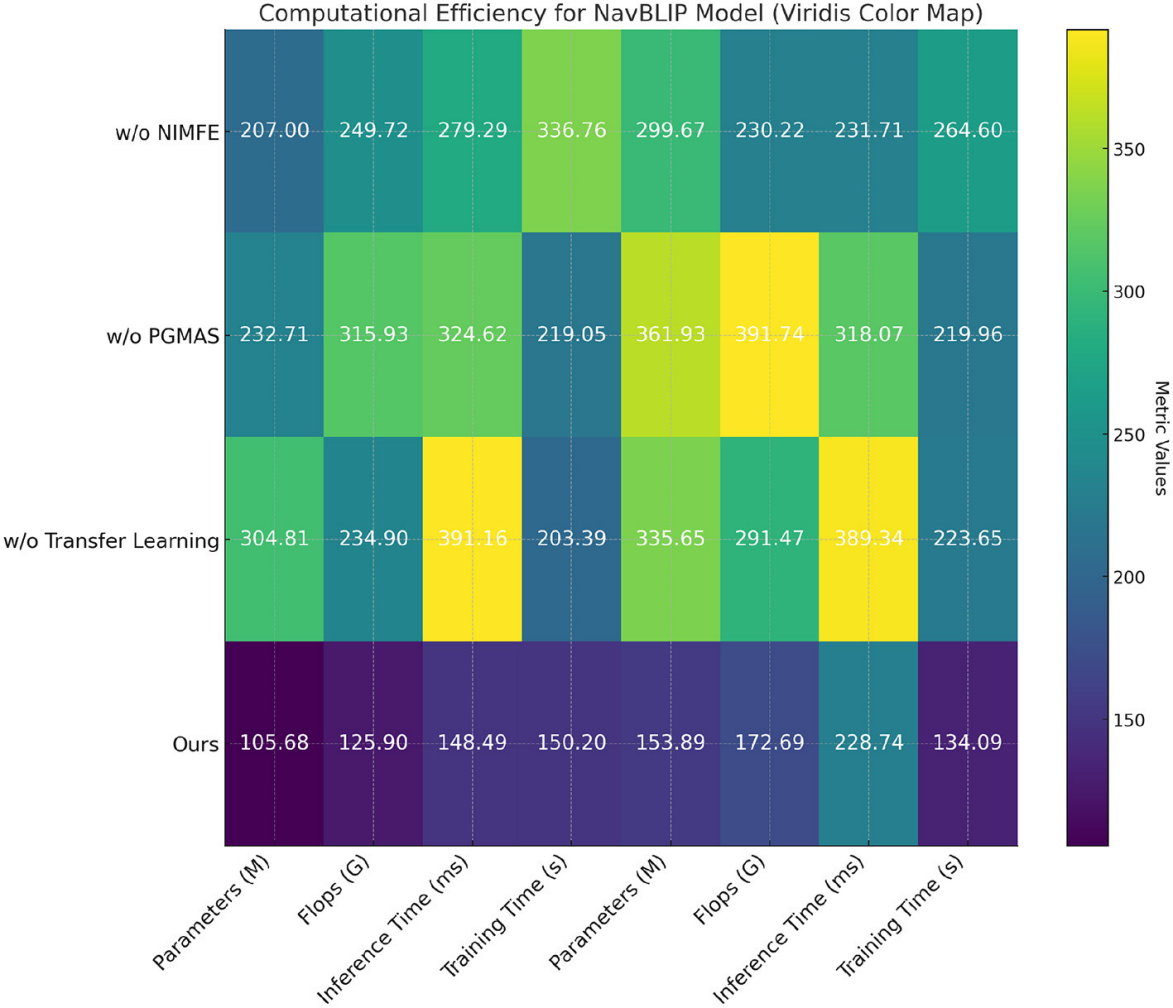
Ablation study: computational efficiency of different components of NavBLIP on RefCOCO and CC12M datasets.

In Table 5 and Figure 6, we evaluate the computational efficiency of NavBLIP and its components through an ablation study on the RefCOCO and CC12M datasets. The full model achieves the best results in terms of both computational efficiency and inference speed. NavBLIP, in its full form, requires 105.68M parameters on the RefCOCO dataset and 153.89M parameters on the CC12M dataset, along with the fewest FLOPs and fastest inference times. When the NIMFE module is removed, the number of parameters increases significantly to 207.00M on RefCOCO and 299.67M on CC12M, indicating that the NIMFE module optimizes the model's capacity by focusing on task-relevant features. Similarly, removing the PGMAS module leads to an increase in FLOPs and training time, further emphasizing the importance of multimodal attention in reducing computational overhead. Removing the transfer learning mechanism also increases the number of parameters and FLOPs, suggesting that transfer learning helps the model leverage pre-trained knowledge and reduces the need for extensive training from scratch. Overall, these results demonstrate that each component of NavBLIP contributes to improving both its computational efficiency and performance, making it suitable for deployment in real-time UAV applications.

**Table 5:** Ablation study: computational efficiency of different components of NavBLIP on RefCOCO and CC12M datasets.

**Method**	**Parameters (M)**	**Flops (G)**	**Inference time (ms)**	**Training time (s)**
**RefCOCO dataset**				
w/o NIMFE	207.00 ± 0.02	249.72 ± 0.02	279.29 ± 0.02	336.76 ± 0.02
w/o PGMAS	232.71 ± 0.02	315.93 ± 0.02	324.62 ± 0.02	219.05 ± 0.02
w/o Transfer learning	304.81 ± 0.02	234.90 ± 0.02	391.16 ± 0.02	203.39 ± 0.02
Ours	**105.68** **±** **0.02**	**125.90** **±** **0.02**	**148.49** **±** **0.02**	**150.20** **±** **0.02**
**CC12M dataset**				
w/o NIMFE	299.67 ± 0.02	230.22 ± 0.02	231.71 ± 0.02	264.60 ± 0.02
w/o PGMAS	361.93 ± 0.02	391.74 ± 0.02	318.07 ± 0.02	219.96 ± 0.02
w/o Transfer learning	335.65 ± 0.02	291.47 ± 0.02	389.34 ± 0.02	223.65 ± 0.02
Ours	**153.89** **±** **0.02**	**172.69** **±** **0.02**	**228.74** **±** **0.02**	**134.09** **±** **0.02**

To validate the necessity of using separate transformations (*f*^(*A*)^, *f*^(*V*)^, *f*^(*W*)^) for each type of nuisance (altitude, view angle, and weather), we conducted a comprehensive ablation study. The objective of this experiment was to assess the impact of separate transformation modules compared to a unified transformation module or no transformation module at all. This study aimed to determine whether treating nuisances independently could provide measurable performance advantages, especially in handling complex environmental conditions. We designed three configurations for this evaluation: (1) Separate Transformation Modules, where each nuisance is processed using a dedicated transformation module tailored to its specific characteristics; (2) Unified Transformation Module, where a single module processes all nuisances collectively; and (3) No Transformation Module, where no explicit nuisance handling is applied. The performance of these configurations was measured on the RefCOCO and CC12M datasets using key metrics including Accuracy, F1 Score, and AUC.

The results, summarized in Table 6, demonstrate the clear benefits of separate transformation modules. The Separate Transformation Modules configuration achieved the highest performance across all metrics. For instance, on the RefCOCO dataset, it recorded an Accuracy of 97.6% and an AUC of 96.7%, while on the CC12M dataset, it achieved an Accuracy of 97.1% and an AUC of 96.5%. This highlights the effectiveness of handling nuisances independently, as it allows the model to better capture nuisance-specific variations and produce robust feature representations. In contrast, the Unified Transformation Module configuration resulted in significantly lower performance, with Accuracy dropping to 94.1% and AUC to 89.5% on the RefCOCO dataset. Similarly, the No Transformation Module configuration performed the worst, with Accuracy reduced to 90.8% on RefCOCO and 90.7% on CC12M. These findings confirm the importance of explicitly addressing nuisance factors and validate our design choice to employ separate transformation modules. Moreover, the cross-modal attention mechanism integrated into our framework further ensures that interactions between nuisances are effectively captured, enhancing the model's adaptability to real-world environments.

**Table 6:** Ablation study results: performance of different transformation settings on RefCOCO and CC12M datasets.

**Setting**	**Dataset**	**Accuracy (%)**	**F1 score (%)**	**AUC (%)**
Separate transformation modules (*f*^(*A*)^, *f*^(*V*)^, *f*^(*W*)^)	RefCOCO	97.6 ± 0.02	93.8 ± 0.03	96.7 ± 0.01
Separate transformation modules (*f*^(*A*)^, *f*^(*V*)^, *f*^(*W*)^)	CC12M	97.1 ± 0.01	92.8 ± 0.03	96.5 ± 0.02
Unified transformation module	RefCOCO	94.1 ± 0.03	86.9 ± 0.02	89.5 ± 0.01
Unified transformation module	CC12M	91.8 ± 0.02	85.9 ± 0.01	86.3 ± 0.03
No transformation module	RefCOCO	90.8 ± 0.01	84.3 ± 0.02	87.0 ± 0.03
No transformation module	CC12M	90.7 ± 0.03	84.6 ± 0.01	86.0 ± 0.02

## 5 Conclusion and discussion

in this work, we aimed to address the challenges of UAV-based navigation and object detection in dynamic environments by proposing NavBLIP, a novel visual-language model that integrates multimodal inputs with advanced feature disentanglement techniques. UAVs often face difficulties due to nuisances such as varying altitudes, weather conditions, and complex object detection tasks. To overcome these challenges, NavBLIP combines the Nuisance-Invariant Multimodal Feature Extraction (NIMFE) module, Prior Guided Multimodal Attention Strategy (PGMAS), and transfer learning techniques to enhance robustness and adaptability. These components allow the model to disentangle task-relevant features from nuisances and effectively integrate metadata such as altitude and weather for better object detection and navigation. The experiments were designed to evaluate the performance of NavBLIP on four diverse datasets: RefCOCO, CC12M, CC3M, and OpenImages. Results demonstrated that NavBLIP consistently outperforms six state-of-the-art models across all key metrics, including accuracy, recall, F1 score, and AUC. The ablation studies further highlighted the importance of each component in improving both model performance and computational efficiency. NavBLIP exhibited significant improvements in inference time and parameter efficiency, making it suitable for real-time UAV applications. However, two notable limitations remain. First, while NavBLIP achieves state-of-the-art performance, it still requires substantial computational resources for training, which may limit its applicability in resource-constrained environments. Second, despite the model's robustness to a variety of conditions, its performance on highly complex, unseen environments could be further improved with more adaptive learning mechanisms. In the future, we plan to explore lightweight versions of NavBLIP, incorporating more efficient model compression techniques to reduce training overhead. Additionally, integrating meta-learning approaches could allow NavBLIP to adapt more quickly to unseen environments, further enhancing its applicability in real-world UAV operations.

## Data Availability

The original contributions presented in the study are included in the article/Supplementary material, further inquiries can be directed to the corresponding author.
