# Supplementary figures and images for: NavBLIP: a visual-language model for enhancing unmanned aerial vehicles navigation and object detection
Source: Front Neurorobot. 2025 Jan 24;18:1513354. doi: 10.3389/fnbot.2024.1513354 (PMC11802496; doi:10.3389/fnbot.2024.1513354)

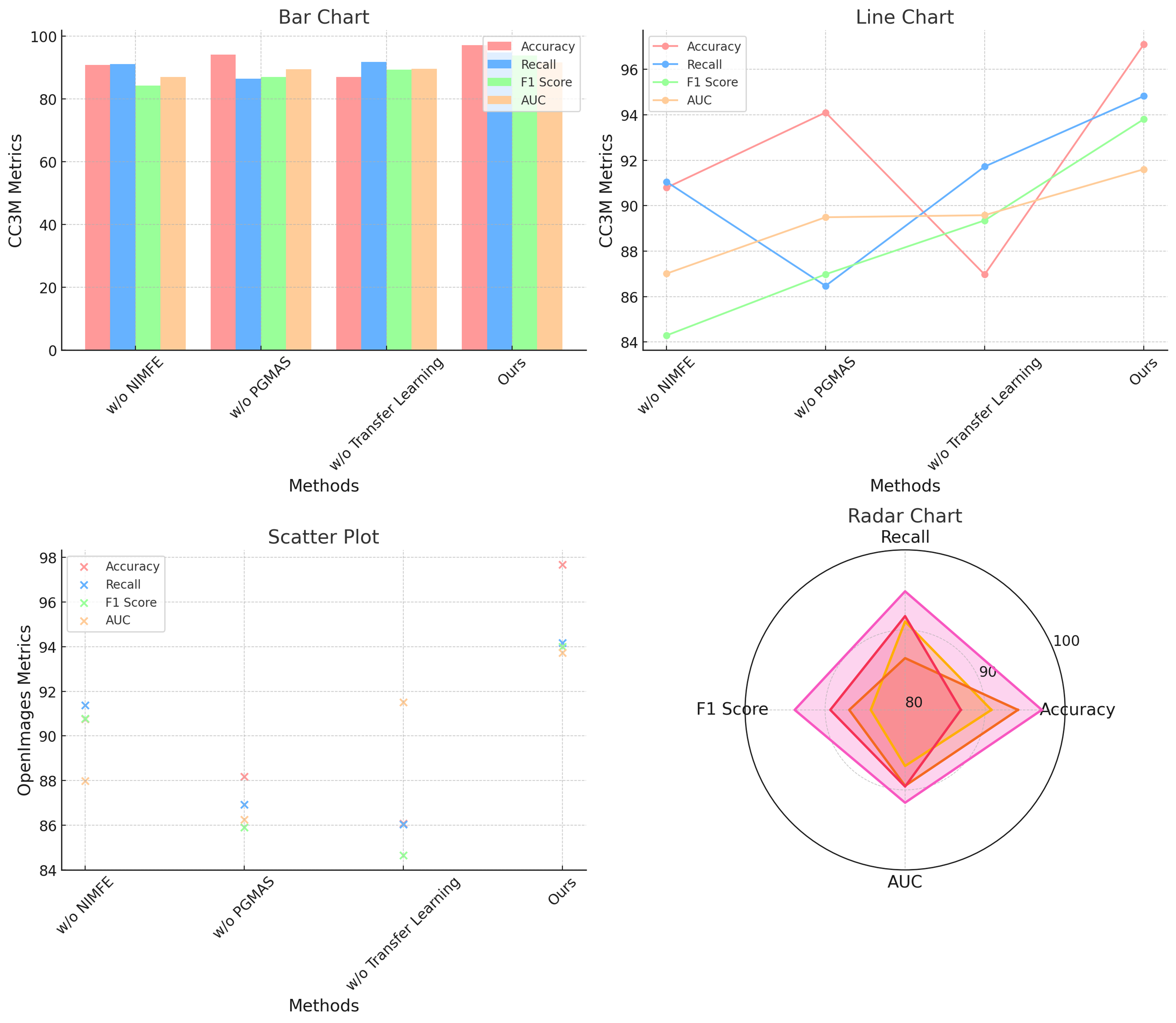

Supplement: Supplementary file 1 [file Image_1.png]

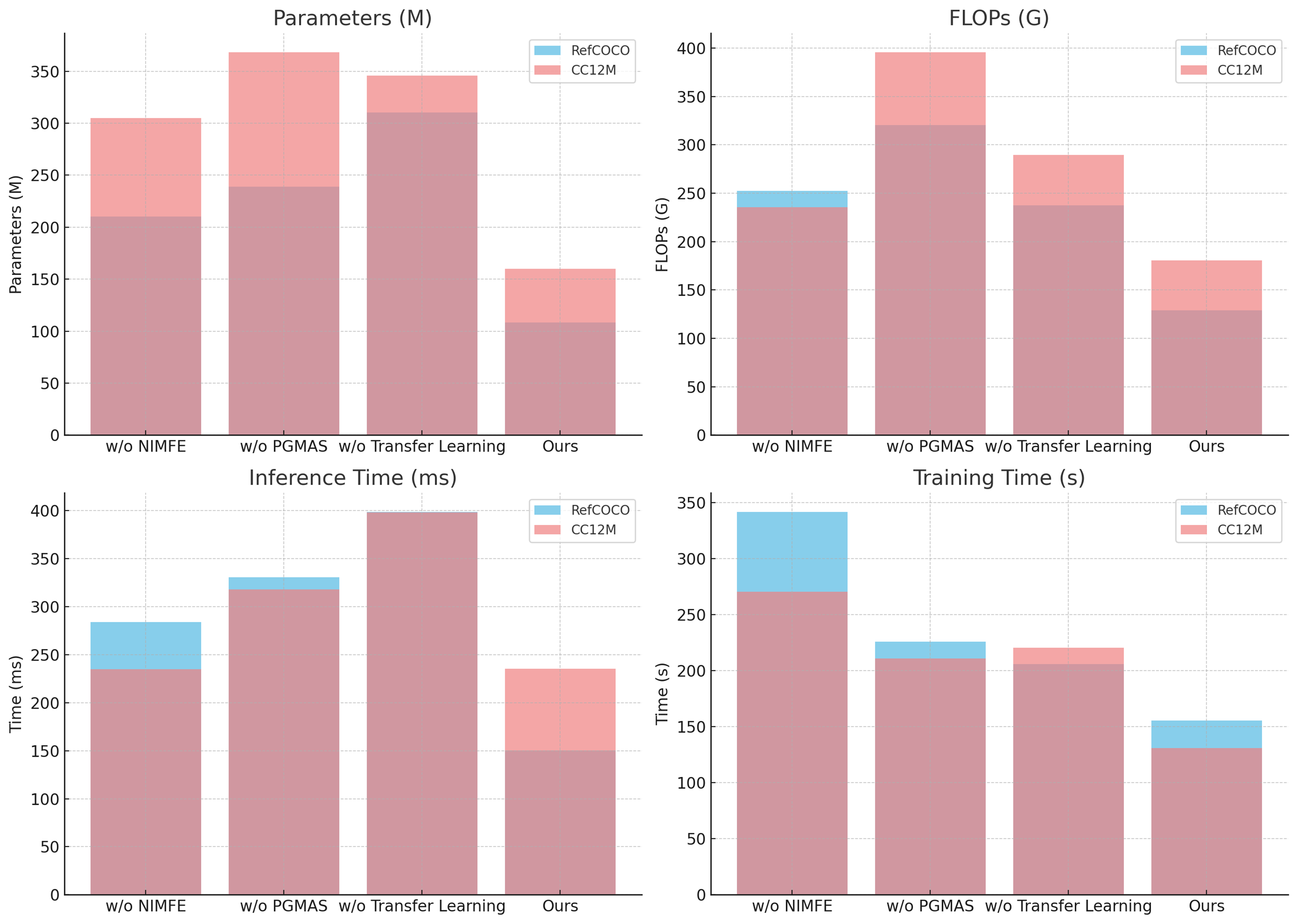

Supplement: Supplementary file 2 [file Image_2.png]
